# Draft Genome Sequence of KCTC 12630, the Type Strain of the Novel Species Sphingomonas ginsengisoli

**DOI:** 10.1128/MRA.01032-18

**Published:** 2019-01-10

**Authors:** Yan-Qiong Li, Hui Zhang, Manik Prabhu Narsing Rao, Zhou-Yan Dong, Dalal Hussien M. Alkhalifah, Wael N. Hozzein, Min Xiao, Wen-Jun Li

**Affiliations:** a Kunming Medical University Haiyuan College, Kunming, People’s Republic of China; b State Key Laboratory of Biocontrol and Guangdong Provincial Key Laboratory of Plant Resources, School of Life Sciences, Sun Yat-Sen University, Guangzhou, People’s Republic of China; c Biology Department, Faculty of Science, Princess Nourah bint Abdulrahman University, Riyadh, Kingdom of Saudi Arabia; d Zoology Department, College of Science, King Saud University, Riyadh, Kingdom of Saudi Arabia; University of Arizona

## Abstract

We report here the draft genome sequence of Sphingomonas ginsengisoli KCTC 12630^T^. The draft genome has a size of 3,045,889 bp and a G+C content of 67.1%. The availability of the genome sequence will provide a better understanding of strain KCTC 12630^T^ and the genus *Sphingomonas*.

## Abstract (precis)

We report here the draft genome sequence of Sphingomonas ginsengisoli KCTC 12630^T^. The draft genome has a size of 3,045,889 bp and a G+C content of 67.1%.

## ANNOUNCEMENT

The genus *Sphingomonas* was first described by Yabuuchi et al. (1), and at the time of writing, the genus comprised 126 recognized species (http://www.bacterio.net/sphingomonas.html) (2). The genus *Sphingomonas* is very important for biotechnological applications due to the ability of the members to produce several bioactive molecules (3–5).

Sphingomonas ginsengisoli was reported by An et al. (6) and was isolated from soil collected in a ginseng field in Pocheon Province, South Korea. Here, we report the draft genome sequence of KCTC 12630, the type strain of the novel species Sphingomonas ginsengisoli, to provide a better understanding of this strain and the genus *Sphingomonas.* Strain KCTC 12630^T^ was cultured on Reasoner’s 2A agar incubated at 30°C. The biomass obtained after 4 days of incubation was collected. The genomic DNA from the biomass for whole-genome sequencing (WGS) was extracted employing the sodium dodecyl sulfate-cetyltrimethylammonium bromide (SDS-CTAB) method (7). Sequencing libraries were generated using the NEBNext Ultra DNA library prep kit for Illumina (New England Biolabs, USA) following manufacturer recommendations. The library quality was assessed on the Qubit version 3.0 fluorometer (Bio-Rad) and Agilent 4200 system. Whole-genome sequencing of strain KCTC 12630^T^ was performed by sequencing paired-end reads on a HiSeq X platform (Illumina, San Diego, CA) at Magigene Company (Guangzhou, People’s Republic of China). The quality of the sequence reads (19,885,796 raw reads and 2,982,869,400 raw bases) was analyzed using the FastQC tool version 0.11.5 (8).

The raw data were filtered (1,923,2872 clean reads and 2,817,875,153 clean bases) using the Fastx-toolkit (9). All good-quality paired reads were assembled using SPAdes version 3.6.0 (10). The draft genome size of strain KCTC 12630^T^ is 3,045,889 bp with an *N*_50_ scaffold length of 1,581,088 bp. The G+C content is 67.1%. Gene prediction was carried out using Glimmer version 3.02 (11). The identification of tRNAs and rRNAs was carried out using tRNAscan-SE version 1.23 (12) and RNAmmer version 1.2 (13). A total of 3,067 genes were predicted with 3 rRNA and 45 tRNA genes. The functional annotation of the genome was performed with Rapid Annotations using Subsystems Technology (RAST) (14), which revealed 182 genes for carbohydrate metabolism and 235 genes involved in protein metabolism.

### Data availability.

The whole-genome sequence reported here has been deposited in GenBank under the accession number QOCK00000000. Raw sequence reads have been deposited in the NCBI Sequence Read Archive under the accession number SRR7938692.
